# Temporal Changes in Air Quality According to Land-Use Using Real Time Big Data from Smart Sensors in Korea

**DOI:** 10.3390/s20216374

**Published:** 2020-11-09

**Authors:** Sung Su Jo, Sang Ho Lee, Yountaik Leem

**Affiliations:** Department of Urban Engineering, Hanbat National University, Daejeon 34158, Korea; gr181203@hanbat.ac.kr (S.S.J.); lshsw@hanbat.ac.kr (S.H.L.)

**Keywords:** smart sensor, real time big data, land-use, air quality, particulate matter (PM10 PM2.5)

## Abstract

This study analyzed the changes in particulate matter concentrations according to land-use over time and the spatial characteristics of the distribution of particulate matter concentrations using big data of particulate matter in Daejeon, Korea, measured by Private Air Quality Monitoring Smart Sensors (PAQMSSs). Land-uses were classified into residential, commercial, industrial, and green groups according to the primary land-use around the 650-m sensor radius. Data on particulate matter with an aerodynamic diameter <10 µm (PM10) and <2.5 µm (PM2.5) were captured by PAQMSSs from September‒October (i.e., fall) in 2019. Differences and variation characteristics of particulate matter concentrations between time periods and land-uses were analyzed and spatial mobility characteristics of the particulate matter concentrations over time were analyzed. The results indicate that the particulate matter concentrations in Daejeon decreased in the order of industrial, housing, commercial and green groups overall; however, the concentrations of the commercial group were higher than those of the residential group during 21:00–23:00, which reflected the vital nighttime lifestyle in the commercial group in Korea. Second, the green group showed the lowest particulate matter concentration and the industrial group showed the highest concentration. Third, the highest particulate matter concentrations were in urban areas where commercial and business functions were centered and in the vicinity of industrial complexes. Finally, over time, the PM10 concentrations were clearly high at noon and low at night, whereas the PM2.5 concentrations were similar at certain areas.

## 1. Introduction

There has been an increase in interest in air quality owing to its effects on the health and quality of life of communities in urban areas [1]. Particularly, the effect of particulate matter influxes to cities from pollutants originating outside the cities [2] and the effect of pollutants from China, such as yellow smog [3], are factors that may amplify particulate matter concentrations in South Korea [4]. Previous studies have reported that particulate matter can have fatal impacts on vulnerable groups, including elderly people, pregnant women, and children, and that it has a close relationship with mortality rates; for instance, in the case of particulate matter with an aerodynamic diameter < 10 µm (PM10), mortality rates from disease increase by 0.3% as the concentration increases by 10 µg/m^3^ [5,6,7,8,9,10]. 

In 2013, the International Agency for Research on Cancer under the World Health Organization (WHO) classified particulate matter as a first-class carcinogen. Accordingly, communities began to pay attention to information on the atmospheric environment (such as particulate matter generated in urban areas), and hence, relevant data were required. Recently, air quality has emerged as the most serious urban and social problem in Korea [11]. As a result, the demand for home appliances, such as air purifiers, has increased rapidly [12]. Smart city plans are being promoted by Korean local governments to address urban problems, such as air quality [13]. In the smart city plan that was presented after 2018, a number of services were proposed to solve the problem of particulate matter [13]. National Air Quality Monitoring Sensors (NAQMSs) were implemented at 502 locations nationwide (as of September 2020) and have been continuously recording atmospheric environmental data, including concentrations of PM10, particulate matter with an aerodynamic diameter < 2.5 µm (PM2.5), O_3_, NO_2_, CO, and SO_2_. The collected data are then provided to the general public through an internet portal in Korea.

The popularization of smart sensors led by the advancement of information and communications technology (ICT) has enabled private companies to promptly provide urban environment data, such as PM10 and PM2.5 concentrations, to communities. An application called ‘Air Map Korea’ is one example. It collects atmospheric environmental data (including of particulate matter) through Private Air Quality Monitoring Smart Sensors (PAQMSSs) from 2400 locations across the country and provides them to the public. PAQMSSs were installed by a Korean private telecommunications company at a location where particulate matter pollution is growing seriously. The purpose of the PAQMSSs project is to measure and provide data for particulate matter at the height of citizen’s breathing [14]. The majority of the nationally operated NAQMSs are located on the roofs of buildings. Considering the spread of particulate matter, it is important to measure the fine dust at the height at which citizens breathe [15]. There are 4.7 times more PAQMSSs than NAQMSs, which comprise 504 sensors across the nation that are managed by the national government.

NAQMSs guarantee reliable atmospheric environmental data; however, their high cost (USD 20,300/sensor) in addition to the difficulty in implementation at multiple locations are limitations of NAQMSs that constrain their range of coverage in urban areas. In contrast, PAQMSSs collect big data on the atmospheric environment across a wider range through affordable smart sensors and provide the data to the public free of charge. 

A total of 12 NAQMSs are located in the city of Daejeon, implying that each sensor covers approximately 45 km^2^ in the entire city area; in the case of urbanized areas, each sensor covers approximately 8 km^2^. Given the results of previous studies that found that dust concentrations varied by land-use [16,17,18,19], NAQMSs do not provide accurate information regarding the air quality of spaces where people live and work. Affordable PAQMSSs (134 sensors) have been implemented by a private company throughout Daejeon and provide more accurate particulate matter information to the public. For instance, each PAQMSS in the entire city of Daejeon covers approximately 4 km^2^, and in urbanized areas, each sensor covers approximately 0.7 km^2^. In the case of urbanized areas in Daejeon, the area covered by PAQMSSs is approximately 11.4 times larger than that covered by NAQMSs. 

Particulate matter research has been conducted from both humanitarian and environmental aspects. Studies in the humanities involve the relationships between, and the implications of, the number of vehicle registrations, industrial locations, traffic facilities, and particulate matter effects [20,21,22,23], the implications of particulate matter according to land-use and seasons [24,25,26], relationships between particulate matter, population density, and traffic volume [27,28,29,30], characteristics of particulate matter concentrations according to transportation, green areas, and building distribution [31,32,33], and changes in particulate matter concentrations on urban heat islands [34,35].

Several studies have been conducted regarding environmental aspects, such as the relationships between particulate matter and weather conditions (such as temperature, wind direction, wind speed, and precipitation) [36,37,38], characteristics of particulate matter concentrations reflecting green area structures and vegetation indices [39,40], and the effects of plants and vegetation in reducing particulate matter [41,42].

The majority of previous studies used statistical methods to analyze particulate matter based on relationships between humanitarian and environmental factors and were conducted using data collected from a limited number of NAQMSs. Although studies have been conducted on spatial aspects, as well as the implications of relationships between particulate matter risks to health, sources of occurrence, humanities, and the environment [18], there have been insufficient studies related to particulate matter distribution using spatial information. Therefore, this study analyzed changes in particulate matter concentrations according to time and land-use and the spatial characteristics of the distribution of particulate matter concentrations according to real-time using big data of PM10 and PM2.5 in Daejeon measured by PAQMSSs. 

The study was conducted from September‒October, (i.e., fall) 2019 in the city of Daejeon, South Korea. Data from September‒October were used for the following reason: particle matter concentrations are relatively lower in Korea from September to October than in other seasons [26,43]. This means that there is little effect of influx of yellow dust from other countries (e.g., China) and variable control was done naturally [43]. Accordingly, it is possible to accurately identify which land-use has the highest impact on particulate matter concentrations. First, five time periods were classified with consideration of human behavior: AM1 (03:00–05:00), AM2 (07:00–09:00), Noon (11:00–13:00), PM1 (17:00–19:00), and PM2 (21:00–23:00). Second, the study determined the mean distance (650-m buffer) with the intention of considering PAQMSS locations and appropriately including areas based on land-use by utilizing a nearest neighbor analysis (NNA). Third, land-uses at locations where PAQMSSs were implemented were classified into four groups: residential, commercial, industrial, and green, according to the land-use ratio based on the 650-m buffer, and k-means clustering was conducted. Next, the differences and variation characteristics of the particulate matter concentrations between time and land-use groups were analyzed using nonparametric test methods, i.e., Kruskal–Wallis test and Mann–Whitney U test. Finally, the inverse distance-weighted method (IDWM) was used to determine the spatial mobility characteristics of particulate matter concentrations over time. 

## 2. Literature Review

Types of particulate matter are determined by their aerodynamic diameter as either PM10 (<10 µm) or PM2.5 (<2.5 µm). The size of PM10 is approximately one-fifth to one-seventh of the diameter of a human hair, whereas PM2.5 is about one-twentieth to one-thirtieth [44]. There are natural and artificial sources of particulate matter, which is defined as invisible dust, including not only solid particles in the air but also smoke emitted from fossil fuels [44]. Examples of natural sources are soil and pollen, and artificial sources are generated from industries and human activities, such as exhaust fumes from cars, tire dust, and crematory fumes [44]. PM2.5 contains SO_2_, NO_2_, CO, and heavy metals and is a secondary pollutant generated when air pollutants, such as sulfur oxides and nitrogen oxides, combine and undergo chemical reactions [44]. 

Studies of air quality related to PM10 and PM2.5 that may have a critical impact on humans have been undertaken. Hwang et al. [16] assessed the status of particulate matter pollution using PM10 data obtained from 11 NAQMSs in the city of Daegu, South Korea from 2006‒2008 and weather data, including wind direction and wind speed. Additionally, in this study, NAQMSs were divided into residence, commerce, industry, and green groups according to the location characteristics, and the implications of weather factors on particulate matter were analyzed depending on the land-use. The results showed that PM10 concentrations in fall and winter were higher than those in spring or summer and that the particulate matter concentrations in industrial areas were twice as high as those in residential areas. In addition, it was reported that particulate matter concentrations would be higher during days without wind and with fog. 

Jeong [22] conducted a spatial distribution analysis using IDWM on the average annual PM10 concentration data collected via NAQMSs from 2000–2005 in Seoul, Korea. The results showed that the particulate matter concentration decreased in the order of winter, spring, fall, and summer and that considerable amounts of PM10 were generated in areas with traffic, dense populations, and large-scale construction sites. In other words, it concluded that particulate matter concentrations were not high across the entire city of Seoul but rather tended to be higher in certain areas.

Jeong and Lee [29] analyzed the particulate matter distribution in Seoul over time, focusing on PM10 and PM2.5 data captured by NAQMSs on the 17th and 18th January 2018. The study used IDWM to identify the relationships between land-use, traffic volume, and particulate matter. Results showed that the distribution of particulate matter concentrations exhibited different spatial and temporal patterns and that commercial areas and traffic increased the particulate matter concentrations, whereas green areas reduced the particulate matter concentrations. 

Jeon et al. [18] conducted an analysis to determine whether there were local differences in the influence of variables on PM10 concentrations, based on the Seoul metropolitan area, using geographically weighted ridge regression and ordinary least squares as research methods. The independent variable was PM10 and the selected dependent variables were natural factors (temperature, precipitation, atmospheric congestion, date, etc.) and human factors (transportation, industrial, residential, commercial, livestock facilities, etc.). The results showed that the lower the precipitation and air movement, the higher the particulate matter concentration. In addition, particulate matter concentrations in livestock or industrial facilities were higher than those in residential or commercial facilities. Overall, the study showed that different factors affected particulate matter concentrations. 

Choi et al. [4] investigated differences in particulate matter concentrations depending on land-use and seasons using PM10 and PM2.5 data collected from NAQMSs in Seoul in 2016. The ratio of the urbanized areas/forest areas located within a 3-km radius of the NAQMSs were divided into three groups; in all cases, the highest PM10 and PM2.5 concentrations occurred in spring and the lowest occurred in summer. Additionally, among the three groups, when the ratio of the forest areas was higher than that of the urbanized areas, particulate matter concentrations were reduced, and this effect was more pronounced in summer than in winter [4].

Choi et al. [26] analyzed the land-use type with the greatest impact on particulate matter using PM10 and PM2.5 data in Seoul in 2016. Based on correlation and regressions, the study reported that particulate matter had a negative correlation with forest areas and a positive correlation with urbanized areas. Moreover, the results showed that broad-leaved forests are more effective in reducing particulate matter than coniferous forests [26].

The preceding studies had the following limitations. First, although it has been shown that particulate matter concentrations differ depending on land-use, the focus has been on interpreting figures, such as statistics, and there remains a lack of studies on temporal and spatial distributions. Second, although NAQMSs enable accurate identification of the widespread generation of particulate matter, they are not densely located, and, hence, further studies using PAQMSSs are required. Given these limitations, this study analyzed changes in particulate matter concentrations according to time and land-use and determined the spatial mobility characteristics of the distribution of particulate matter concentrations using PM10 and PM2.5 big data of particulate matter in Daejeon measured by PAQMSSs. 

## 3. Data and Method

This study utilized PM10 and PM2.5 concentration data measured by PAQMSSs that collect and manage data from 134 locations in Daejeon, from September‒October 2019. Among them, data collected by 123 PAQMSSs were used; 11 PAQMSSs were excluded because missing values were identified due to data transmission errors, etc. The data did not satisfy the normality test, and the total number of data points was 108,072.

The results of basic statistical analysis, including the maximum, minimum, and mean values, are summarized in Table 1. The PAQMSSs (134 locations) operated in Daejeon secured approximately 12 times more branches than the NAQMSs (10 locations). This indicated that PAQMSSs should be used to analyze changes in the PM10 and PM2.5 concentrations in more detail across the entire city of Daejeon. Existing studies show that the data generated by PAQMSSs are as reliable as the nationally-managed NAQMSs [45]. In this study, it was verified that there was no difference between NAQMSs and PAQMSSs data using paired samples t-test. Therefore, this study secured the reliability of the data.

**Figure 1:**
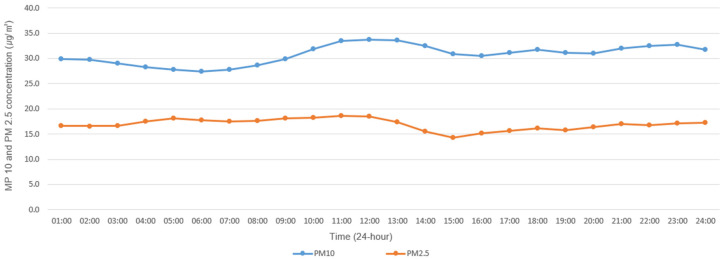
Mean concentrations of particulate matter (PM10, PM2.5) over time obtained from Private Air Quality Monitoring Smart Sensors (PAQMSSs) in Daejeon.

Figure 1 shows the mean particulate matter concentration over time using data from 123 PAQMSSs to identify the trends of the particulate matter concentrations during fall (September‒October). The PM10 concentrations exhibited a pattern of being low at dawn, increasing during the afternoon, and then decreasing in the evening. Particularly, concentrations were highest during Noon (11:00–13:00) and slightly increased after 21:00. PM2.5 showed a similar pattern to PM10 but with less deviation.

The analysis methods used in this study were NNA, k-means clustering, Kruskal–Wallis test, Mann–Whitney U test, and IDWM. NNA was undertaken to consider the distances between each PAQMSS and set a buffer for calculation of the optimum land-use ratio focusing on PAQMSSs. The k-means clustering method was introduced to classify PAQMSSs into four groups according to characteristics: residence, commerce, industry, and green. Cluster analysis using big data can be classified into supervised learning-based K-Nearest Neighbor (KNN) and unsupervised learning-based k-means clustering. This study used k-means clustering based on unsupervised learning because it was determined to be more suitable in this study to classify clusters based on the characteristics of each datum (unsupervised basis). This method involves dividing land-use ratios resulting from each PAQMSS into k groups, with the limitation of estimating the optimal number of k. In this study, k was divided into four groups based on land-use. 

Nonparametric test methods (Kruskal–Wallis test) were used for statistical verification of concentration differences between the five time periods (AM1, AM2, Noon, PM1, and PM2) and land-use groups (residential, commercial, industrial, and green). After the differences between groups and time periods were statistically verified, the Mann–Whitney U test was used to verify differences between groups within the same time period. This is a nonparametric test method that can test PM10 and PM2.5 concentration differences between detailed groups. The significance of the Mann–Whitney U test was determined by the significance level of correction by Bonferroni correction and Kruskal–Wallis tests, and the Mann–Whitney U test was used when data did not satisfy normality. 

PAQMSS data points expressing PM10 and PM2.5 concentrations were plotted on a map using IDWM. IDWM is a method of inversely weighting distances from observation points, wherein a lower weight indicates a larger distance [46]. This study used IDWM to identify regional differences in particulate matter concentrations. Spatial interpolation methods such as kriging and spline using statistical methods exist; however, this study used IDWM due to the lack of normality of the data [47,48].

## 4. Results and Discussion

### 4.1. Classification of Land-Use Group around PAQMSSs

**Figure 2:**
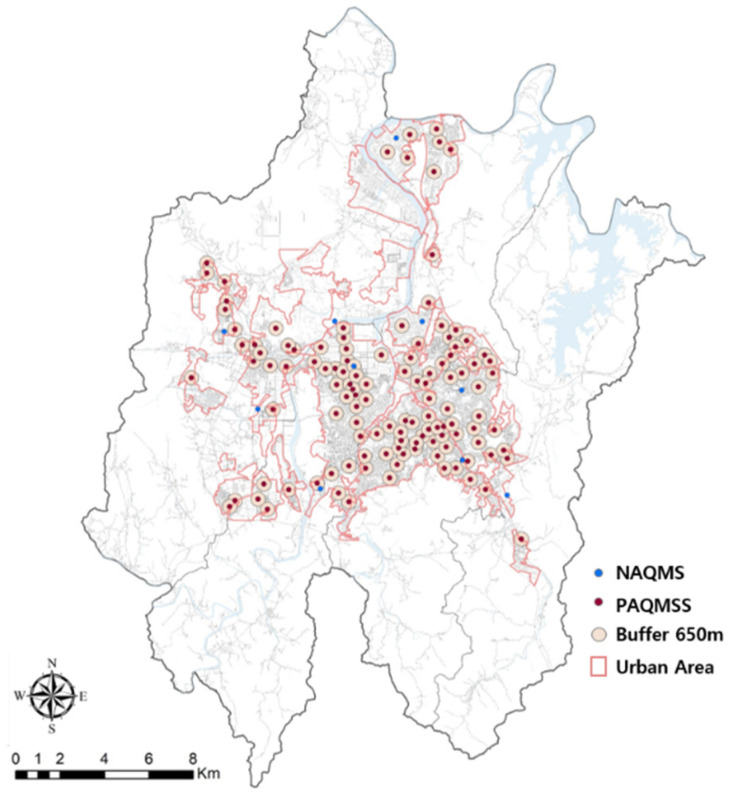
Air Quality Monitoring Sensors (AQMSs) map with 650-m buffer.

The land-use ratio of the area surrounding PAQMSSs depends on the buffer range. In this study, the mean distance between the PAQMSSs was calculated using NNA; therefore, land-use area ratios were appropriately included while considering each PAQMSS’ location and the corresponding distances. The calculated distance among PAQMSSs was derived as the 650-m-radius buffer, taking into account the minimum and maximum distances of PAQMSSs (Figure 2). 

**Figure 3:**
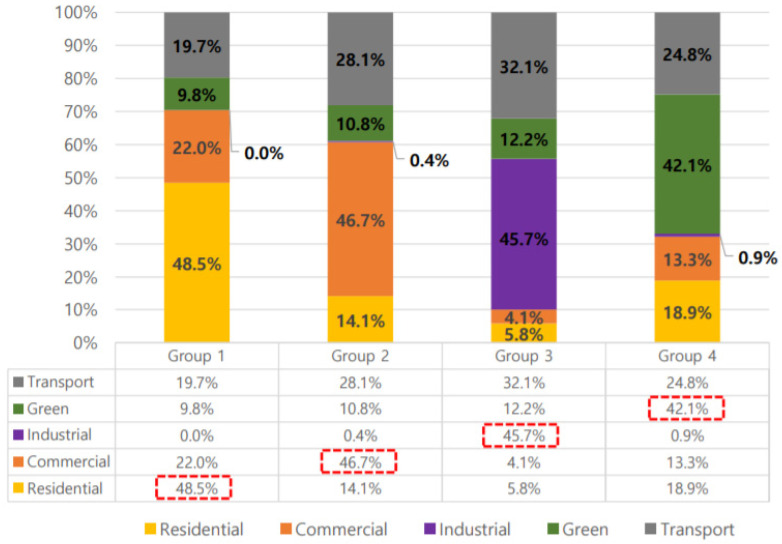
Residential, commercial, industrial, and green area ratio by groups.

**Figure 4:**
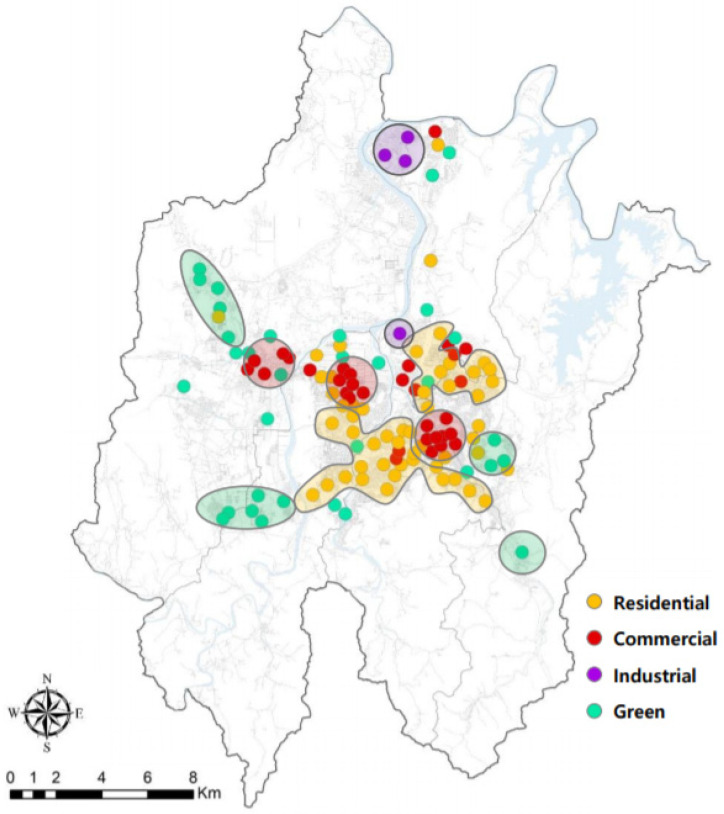
Map of clustered Private Air Quality Monitoring Smart Sensors (PAQMSSs).

A 650-m-diameter buffer centered on PAQMSSs covered > 30.2% of the urbanized areas in Daejeon, i.e., the area covered is 12.6 times larger than that covered by National Air Quality Monitoring Sensors (NAQMSs). Land-use types within the range of the 650-m-diameter buffer were simplified into residence, commerce, industry, green, and roads, and their ratios were determined as 30.5%, 26.3%, 2.7%, 19.8%, and 24.7%, respectively (Table 2). Residential areas accounted for the largest proportion, followed by commercial areas, roads, green areas, and industrial areas. K-means clustering analysis was used to analyze the land-use characteristics of 123 PAQMSSs based on the land-use area ratio and classified the 123 PAQMSSs into four groups: Group 1, Group 2, Group 3, and Group 4 (Figure 3 and Figure 4; Table 3).

The characteristics of PAQMSSs were defined by selecting the largest land-use from the areas within a 650-m diameter from the PAQMSSs. For example, PAQMSSs with a residential area of 100 m^2^, commercial area of 30 m^2^, industrial area of 35 m^2^, green area of 8 m^2^, and transport area of 15 m^2^ within a 650-m diameter belonged to the residential group because the residential area was larger than that of other areas. Each group was defined in this way as a residential, commercial, industrial and green group. Group 1 included 52 PAQMSSs with the largest proportion of residences (48.5%); therefore, it had residential characteristics. Group 2 included 33 PAQMSSs with the largest proportion of commerce (46.7%) and, therefore, was classified as having commercial characteristics. Group 3 included four PAQMSSs and its industrial ratio was 45.7%. This group had industrial characteristics and a higher road ratio than other groups. In Group 4, green areas accounted for the largest percentage (42.1%) and 34 PAQMSSs were included; accordingly, this group was classified as having green characteristics. In this manner, the highest land-use ratio was defined as the characteristic of the group. In terms of characteristics per group, Groups 1, 2, and 4 had the lowest industrial ratios and Group 3 included the lowest residential and commercial ratios. For the green ratio, all groups except Group 4 had low ratios. Group 3 the highest ratio for roads and Group 1 had the lowest. 

The spatial distribution of PAQMSSs included in the four groups is shown in Figure 3. PAQMSSs classified as residential groups were widely distributed across Daejeon’s urbanized areas (yellow dots). Commercial groups of PAQMSSs were concentrated in old and new urban areas that may be considered as core areas in Daejeon (red dots). Industrial groups were located around industrial complexes and were classified as representative industrial areas within the urbanized areas (green dots), and green groups were located on the outskirts of the urbanized areas in Daejeon (Figure 4). The classification of land-use groups through the k-means clustering method and the result of the PAQMSSs distribution chart well-reflected the group characteristics when compared to the current land-use in Daejeon. 

### 4.2. Changes in Particulate Matter Concentrations according to Land-Use and Time Period 

Table 4 and Table 5 present the differences in PM10 and PM2.5 concentrations over time and between land-use groups and the differences in concentrations between groups within the same time frame. In the cases when the PM10 and PM2.5 concentrations differed between land-use groups over time in the nonparametric test, an additional analysis of the differences between groups within the same time frame was undertaken using the Mann–Whitney U test. In addition, this study used IDWM to visualize and analyze the spatial distribution characteristics of PM10 and PM2.5 concentrations within the regions.

The mean PM10 concentrations from September‒October in Daejeon were moderate (26.4 ± 12.3 µg/m^3^~35.8 ± 13.5 µg/m^3^) according to the WHO (Table 4). There were significant differences in the PM10 concentrations between all land-use groups within the same time period (*p* < 0.0083). 

The PM10 concentrations between land-use groups (residence, commerce, industry, and green) showed differences in all time periods (*p* < 0.05). The PM10 concentrations were the lowest in the green group, and the concentrations were high in the order of industrial, residential, and commercial. However, the PM10 concentration during PM2 was 1.2 µg/m^3^ higher in the commercial group than in the residential group (Table 4). 

The industrial and green groups showed the largest differences in PM10 concentration, with differences of 12.5% (AM1), 18.4% (AM2), 26.5% (Noon), 23.7% (PM1), and 18.8% (PM2). PM10 concentrations showed the biggest difference during Noon and the smallest difference during AM1. The PM10 concentrations of the green group (with high forest ratios) were low and the concentrations of the industrial group (with PM10 emission sources, e.g., industry and roads) were high, indicating that the particulate matter concentrations varied depending on the land-use ratio [4]. The land-use groups presenting the smallest differences were the residential and commercial groups; differences between these groups were 0.7% (AM1), 3.0% (AM2), 1.5% (Noon), 0.3% (PM1), and −3.7% (PM2). Unlike the differences between industrial and green groups, the PM10 concentrations between residential and commercial groups had the biggest difference during PM2 and the smallest difference during PM1. 

**Figure 5:**
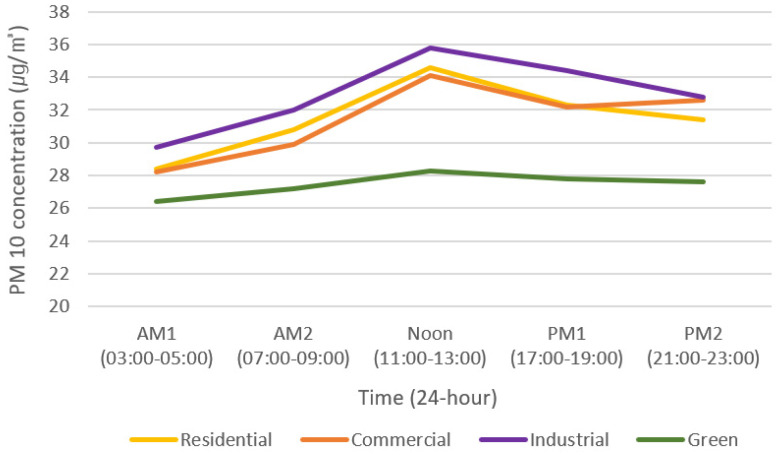
Differences in PM10 concentration by land-use over time.

In particular, it is believed that the commercial group had higher PM10 concentrations than the residential group during PM2 owing to the increased human activities in commercial areas. Industrial groups had higher PM10 concentrations than other groups due to the greater amount of fuel used in industrial areas [43]. The PM10 concentration patterns showed that the concentrations in the residential, commercial, and industrial groups gradually decreased after reaching the peak during Noon (Figure 5). This was understood to be because most of the activities in a city (such as vehicle operation and movement of people) are carried out during the day. 

Except for the industrial group, the land-use groups with large green area ratios showed lower PM10 concentrations. Moreover, as residential area ratios were high, the particulate matter concentrations were characterized to be high, and the PM10 concentrations decreased as green area ratios increased. 

**Figure 6:**
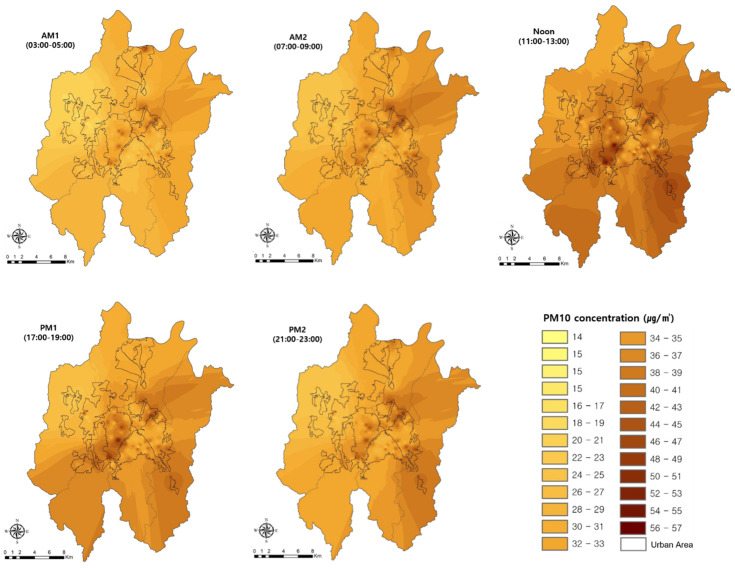
Changes in spatial distribution characteristics of PM10 concentration over time. AM1, AM2, Noon, PM1, and PM2.

The spatial distribution changes in the PM10 and PM2.5 concentrations were analyzed using IDWM (Figure 6). The PM10 concentrations in Daejeon were high in the central area where commercial and business functions were concentrated and in old and new urban areas. Furthermore, the PM10 concentrations were high in the industrial areas where industrial complexes were located. The PM10 concentration gradually began to increase from the northeast over time and spread throughout Daejeon during Noon. Subsequently, it showed a gradually decreasing distribution of the concentrations from the southwest (Figure 6). 

High PM10 concentrations were maintained in the central area where commercial and business functions were concentrated and in the industrial area where industrial complexes were located. The mean PM2.5 concentration was moderate (14.5 ± 6.6 µg/m^3^~21.8 ± 6.4 µg/m^3^), similar to that of PM10. 

Analyses of the PM2.5 concentration changes provided the following results: there were differences in the PM2.5 concentrations between the land-use groups at all times and land-use groups at the same time (*p* < 0.05, *p* < 0.0083). The PM2.5 concentrations were the lowest in the green group for all time periods, and similarly to PM10, they were high in the order of industrial, residential, and commercial. However, the PM10 concentrations during PM2 were 0.5 µg/m^3^ higher in the commercial group than in the residential group. 

The industrial and green groups showed the biggest differences in PM2.5 concentration. The differences between the two groups were 14.8% (AM1), 19.4% (AM2), 21.7% (Noon), 43.4% (PM1), and 36.6% (PM2). The PM2.5 concentration showed the largest differences during PM1 and the smallest differences during AM2, which differed from the results of PM10. This is determined to be a phenomenon in which pollutants (PM10) generated in industrial areas combine with surrounding O_3_ water vapor, resulting in higher PM2.5 concentrations. This was influenced by the fact that all industrial areas in Daejeon are located near rivers [49].

**Figure 7:**
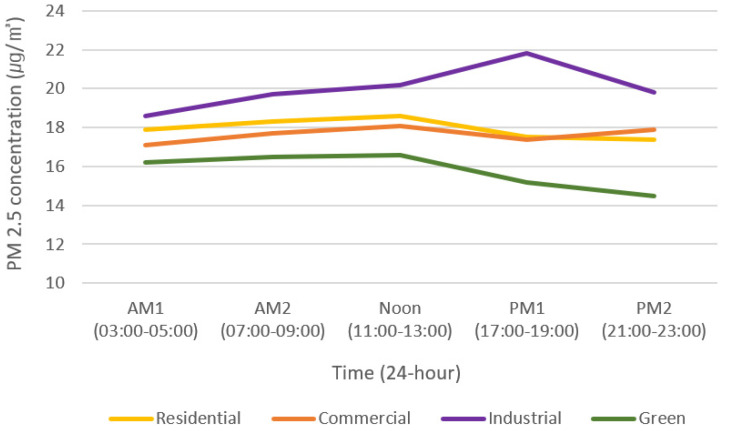
Changes in PM2.5 concentration by land-use group over time.

PM2.5 concentration patterns were similar to those of PM10; however, a constant PM2.5 concentration was characteristically maintained in the residential and commercial groups (Figure 7). In addition, the industrial group showed a phenomenon of peaking during PM1, and the green group showed a steeply declining pattern after Noon. Characteristically, a phenomenon was observed whereby the PM2.5 concentration of the commercial group was higher than that of the residential group during PM2, which was the same pattern as PM10. This is due to the greater movement and energy consumption of vehicles and people in the commercial group than in the residential group during PM2 [29]. 

The residential and commercial groups had the smallest differences in PM 2.5 concentrations, which were 4.7% (AM1), 3.4% (AM2), 2.8% (Noon), 0.6% (PM1), and −2.8% (PM2). The difference in the PM2.5 concentrations between residential and commercial groups was the largest during AM1 and the smallest during PM1. Different from the PM10 results, PM2.5 concentrations were higher at dawn, which indicates the PM2.5 is not easily resolved overnight. 

**Figure 8:**
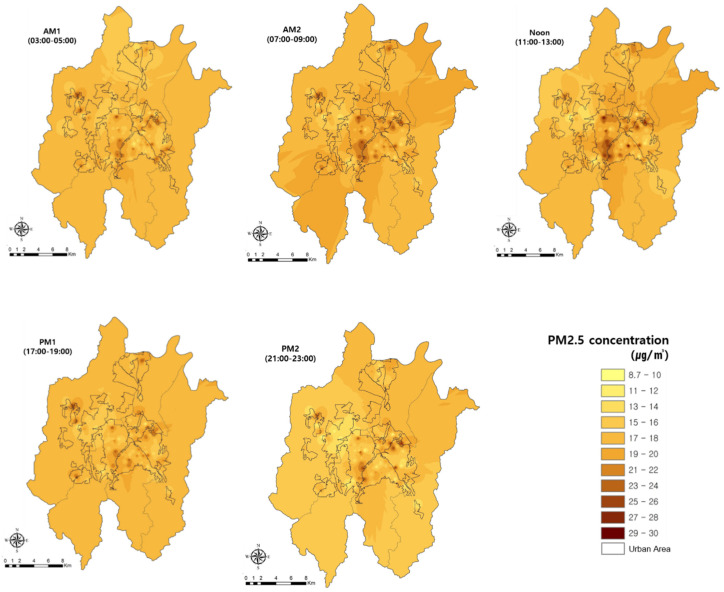
Changes in spatial distribution characteristics of PM2.5 concentrations over time. AM1, AM2, Noon, PM1, and PM2.

The spatial distribution of PM2.5 was similar to that of PM10, whereby the highest concentrations occurred where commercial and business functions were concentrated (Figure 8). However, a constant PM2.5 concentration was maintained at certain locations. Its features were evident in the commercial and industrial groups, i.e., the PM2.5 concentrations were the highest during Noon. Moreover, PM2.5 concentrations were maintained at specific locations, rather than being widely distributed overall. In the central and industrial areas, PM2.5 concentrations were high regardless of time, and the distribution of concentrations had similar characteristics to those of PM10. 

## 5. Conclusions

This study analyzed changes in particulate matter concentrations and the spatial characteristics of the distribution of those concentrations according to time and land-use, using PM10 and PM2.5 big data measured in Daejeon by a private company’s PAQMSSs from September‒October 2019. The results are summarized as follows: first, the land-use types within the range of 650-m-diameter buffers based on 123 PAQMSSs were simplified to residences, commerce, industry, green, and roads, with ratios of 30.5%, 26.3%, 2.7%, 19.8%, and 24.7%, respectively. According to the grouping based on the ratios, four groups (residence, commerce, industry, and green) were classified. Analyses of the highest land-use ratio in each group identified residence (48.5%) in Group 1, commerce (46.7%) in Group 2, industry (45.7%) in Group 3, and green (42.1%) in Group 4. Then, the highest land-use ratio in each group was defined as being characteristic of the group. 

Second, the PM10 and PM2.5 data showed moderate levels of particulate matter concentrations, and there were significant differences in the concentrations between groups over time and between groups at the same time (with the exception of during PM2). Particulate matter concentrations were high during all time periods in the order of the industry, residence, commerce, and green (with the exception of during PM2); however, concentrations in the industrial group were higher than those in the residential group. This may be a result of the increasing mixed land-use due to intensive zoning control. The weakening of zoning control has resulted in a large supply of residential areas in commercial areas, which has caused an increase in nighttime activities.

Third, the groups presenting the biggest concentration differences were both PM10 and PM2.5 in the residential and green groups. In addition, the particulate matter concentration in the green group (with high forest ratios) was low, and the concentration in the industrial group (with high industrial and road ratios) was high. This indicates that the concentrations varied depending on the land-use ratio, which is in agreement with previous studies. Industrial areas use more fuel and have higher emissions of pollutants from combustion facilities and production processes than commercial and residential areas [49]. Moreover, PM10 showed the biggest differences during Noon, whereas PM2.5 showed differences during PM1. The reason for this difference is believed to be that PM10 combined with O_3_ and water vapor and was transformed to PM2.5 by chemical reactions. 

Fourth, PM10 and PM2.5 concentrations tended to be high in old and new urban areas (where commercial and business functions were concentrated), and where the industrial complex was located. Moreover, overall, the particulate matter concentrations were low in the morning (AM1 and AM2), highest in the afternoon (Noon), and gradually increased in the evening (PM1 and PM2). PM10 concentrations clearly showed variations over time, whereas the PM2.5 concentrations had distribution characteristics that remained stable in certain areas. The results of this study show that the PM10 concentration can be resolved naturally over time, however PM2.5 showed a stagnation phenomenon, whereby it was not easily diluted naturally from concentrated areas [50,51]. Addressing this problem involves promoting a long-term policy to reduce the occurrence of particulate matter pollution and, simultaneously, providing physical measures, such as parks and green areas, to minimize the effects of particulate matter on the human body. In other words, firstly, in order to prevent this phenomenon, a policy that minimizes air pollutants is needed. Secondly, green space is important for particulate matter management, as revealed in previous studies [26]. Sufficient parks and green areas should be provided to absorb fine dust generated in industrial, commercial, and residential areas.

This study examined whether particulate matter concentrations changed depending on time and land-use and analyzed the characteristics of the spatial distribution of particulate matter. The residential, commercial, and industrial areas representing urbanized areas were found to increase the particulate matter concentration and the green area was identified as a factor in decreasing the concentration [29,52]. This study provides guidelines for establishing particulate matter reduction policies since environmental policies are significant for pollution reduction. The limitation of the study was that the analysis only considered changes in the particulate matter concentrations in the city of Daejeon. However, the locations of sources of particulate matter vary and the various causes are complex and interwoven. Therefore, studies on a range of aspects are required, including the degree of influence of particulate matter between areas and the causes of occurrence, by expanding the current research scope. 

## Figures and Tables

**Table 1:** Descriptive statistics of PM10 and PM2.5 (unit: µg/m^3^).

Type of Particulate Matter	No. of PAQMSSs	n	Concentration				
			Min.	Max.	Mean	SD	Variance
PM10	123	180,072	14.75	62.32	31.89	5.40	54.71
PM2.5	123	180,072	8.71	32.68	16.72	3.85	27.75

**Table 2:** Land-use ratio and area in 650-m buffer (unit: m^2^).

	Residential	Commercial	Industrial	Green	Transport
Min.	0.0	4653.5	0.0	0.0	9866.1
	(0.0%)	(1.2%)	(0.0%)	(0.0%)	(3.4%)
Max.	274,022.4	252,028.1	254,441.7	263,251.6	178,822.2
	(83.1%)	(76.5%)	(77.1%)	(79.6%)	(54.2%)
Mean	98,948.3	84,881.1	6114.2	63,022.8	78,729.0
	(30.5%)	(26.3%)	(2.7%)	(19.8%)	(24.7%)
SD	63,844.4	54,864.4	29,954.9	57,243.1	32,028.7
	(19.4%)	(17.5%)	(9.2%)	(17.7%)	(10.5%)

**Table 3:** Results of k-means clustering analysis.

	Classification of Clustering				F	*p*-Value
	Group 1 (*n* = 52)	Group 2 (*n* = 33)	Group 3 (*n* = 4)	Group 4 (*n* = 34)		
Residential	0.485	0.141	0.058	0.189	104.155	0.000
Commercial	0.220	0.467	0.041	0.133	79.290	0.000
Industrial	0.000	0.004	0.457	0.009	155.736	0.000
Green	0.098	0.108	0.122	0.421	86.732	0.000
Transport	0.197	0.281	0.321	0.248	7.422	0.000
Group Characteristics	Residential Group	Commercial Group	Industrial Group	Green Group	-	

**Table 4:** Differences in PM10 concentration by land-use over time.

Period	Group	PM10 (µg/m^3^) Mean ± SD	Kruskal–Wallis	Paired Comparison					
				Residential–Commercial	Residential–Industrial	Residential–Green	Commercial–Industrial	Commercial–Green	Industrial–Green
AM1 (03:00‒05:00)	Residential	28.4 ± 14.4	χ^2^ = 31.104 *p* = 0.001 *	Z = −3.620 *p* = 0.000 **	Z = −1.417 *p* = 0.000 **	Z = −5.314 *p* = 0.000 **	Z = −0.367 *p* = 0.002 **	Z = −1.327 *p* = 0.005 **	Z = −1.175 *p* = 0.000 **
	Commercial	28.2 ± 11.9							
	Industrial	29.7 ± 10.0							
	Green	26.4 ± 12.3							
AM2 (07:00‒09:00)	Residential	30.8 ± 14.9	χ^2^ = 20.490 *p* = 0.001 *	Z = −3.278 *p* = 0.001 **	Z = −1.560 *p* = 0.001 **	Z = −4.086 *p* = 0.000 **	Z = −0.016 *p* = 0.001 **	Z = −0.641 *p* = 0.001 **	Z = −0.362 *p* = 0.001 **
	Commercial	29.9 ± 12.7							
	Industrial	32.2 ± 9.7							
	Green	27.2 ± 13.4							
Noon (11:00‒13:00)	Residential	34.6 ± 17.0	χ^2^ = 29.267 *p* = 0.001 *	Z = −1.923 *p* = 0.004 **	Z = −5.118 *p* = 0.001 **	Z = −0.323 *p* = 0.004 **	Z = −4.093 *p* = 0.000 **	Z = −1.967 *p* = 0.001 **	Z = −4.945 *p* = 0.000 **
	Commercial	34.1 ± 16.8							
	Industrial	35.8 ± 13.5							
	Green	28.3 ± 27.3							
PM1 (17:00‒19:00)	Residential	32.3 ± 18.2	χ^2^ = 26.899 *p* = 0.001*	Z = −3.975 *p* = 0.000 **	Z = −3.950 *p* = 0.000 **	Z = −2.649 *p* = 0.001 **	Z = −1.894 *p* = 0.000 **	Z = −1.218 *p* = 0.003 **	Z = −2.541 *p* = 0.000 **
	Commercial	32.2 ± 17.2							
	Industrial	34.4 ± 14.5							
	Green	27.8 ± 18.5							
PM2 (21:00‒23:00)	Residential	31.4 ± 14.7	χ^2^ = 23.205 *p* = 0.001 *	Z = −3.667 *p* = 0.000 **	Z = 1.580 *p* = 0.000 **	Z = −4.246 *p* = 0.000 **	Z = −0.156 *p* = 0.001 **	Z = −0.395 *p* = 0.001 **	Z = −0.362 *p* = 0.000 **
	Commercial	32.6 ± 12.6							
	Industrial	32.8 ± 11.1							
	Green	27.6 ± 12.4							

* *p* < 0.05, ** *p* < 0.0083 (Adjusted by Bonferroni correction method = 0.05/6).

**Table 5:** Differences in PM 2.5 concentration by land-use over time.

Period	Group	PM2.5 (µg/m^3^) Mean ± SD	Kruskal– Wallis	Paired Comparison					
				Residential – Commercial	Residential – Industrial	Residential – Green	Commercial – Industrial	Commercial – Green	Industrial – Green
AM1 (03:00‒05:00)	Residential	17.9 ± 8.6	χ^2^ = 32.139 *p* = 0.000 *	z = −3.451 *p* = 0.001 **	Z = −0.251 *p* = 0.002 **	Z = −5.425 *p* = 0.000 **	Z = −1.318 *p* = 0.001 **	Z = -−1.576 *p* = 0.001 **	Z = −2.185 *p* = 0.000 **
	Commercial	17.1 ± 10.2							
	Industrial	18.6 ± 7.7							
	Green	16.2 ± 8.6							
AM2 (07:00‒09:00)	Residential	18.3 ± 8.3	χ^2^ = 29.273 *p* = 0.000 *	Z = −3.282 *p* = 0.001 **	Z = −2.692 *p* = 0.007 **	Z = −4.943 *p* = 0.000 **	Z = −1.125 *p* = 0.001 **	Z = −1.344 *p* = 0.000 **	Z = −0.0447 *p* = 0.000 **
	Commercial	17.7 ± 9.5							
	Industrial	19.7 ± 7.7							
	Green	16.5 ± 9.2							
Noon (11:00‒13:00)	Residential	18.6 ± 7.1	χ^2^ = 5.041 *p* = 0.000 *	Z = −1.603 *p* = 0.001 **	Z = −1.656 *p* = 0.000 **	Z = −0.047 *p* = 0.000 **	Z = −0.831 *p* = 0.000 **	Z = -−1.427 *p* = 0.001 **	Z = −1.502 *p* = 0.000 **
	Commercial	18.1 ± 8.2							
	Industrial	20.2 ± 6.5							
	Green	16.6 ± 7.9							
PM1 (17:00‒19:00)	Residential	17.5 ± 7.2	χ^2^ = 39.001 *p* = 0.000 *	Z = −3.253 *p* = 0.001 **	Z = −1.556 *p* = 0.000 **	Z = −5.510 *p* = 0.000 **	Z = −2.942 *p* = 0.003 **	Z = −1.880 *p* = 0.000 **	Z = −3.994 *p* = 0.000 **
	Commercial	17.4 ± 8.6							
	Industrial	21.8 ± 6.4							
	Green	15.2 ± 7.9							
PM2 (21:00‒23:00)	Residential	17.4 ± 6.7	χ^2^ = 23.195 *p* = 0.000 *	Z = −2.718 *p* = 0.007 **	Z = −2.207 *p* = 0.001 **	Z = −4.550 *p* = 0.000 **	Z = −0.947 *p* = 0.001 **	Z = −1.436 *p* = 0.000 **	Z = −0.203 *p* = 0.000 **
	Commercial	17.9 ± 7.9							
	Industrial	19.8 ± 6.7							
	Green	14.5 ± 6.6							

* *p* < 0.05, ** *p* < 0.0083 (adjusted by Bonferroni correction method = 0.05/6).
